# The Correlation Between Justice and Organizational Citizenship Behavior and Organizational Identity Among Nurses 

**DOI:** 10.5539/gjhs.v6n6p252

**Published:** 2014-08-15

**Authors:** Arbabisarjou Azizollah, Reza Hajipour, Sadeghian Sourki Mahdi

**Affiliations:** 1 Pregnancy Health Research Center, Zahedan University of Medical Sciences, Zahedan, Iran; 2 Faculty of Social Sciences, Islamic Azad University Dehaghan Branch, Iran; 3 Faculty of Education, University of Isfahan, Isfahan, Iran

**Keywords:** organizational citizenship behavior, organizational identity, nurses, justice

## Abstract

“The correlation between justice and organizational citizenship behavior and organizational identity among the nurses”, aimed to correlate different aspects of personal feelings and organizational identity in a population of nurses. The population included all nurses working at hospitals affiliated to administry of health, treatment and medical education in Shahre-Kord (Iran) 2009. A sample consisting of 168 nurses was randomly selected out of the population. The study adopted a descriptive-correlative method. The Organizational Justice Questionnaire (1998), the Organizational Citizenship Questionnaire, and Organizational Identity Questionnaire (1982) were used for gathering data. Data was analyzed through multiple regression analysis. The findings revealed that 4 dimensions of organizational citizenship behavior (altruism, civic virtue, conscientiousness, and self-development) are correlated with organizational identity (R^2^ = 0.612); and loyalty and obedience are correlated with distributional justice (R^2^ = 0.71). Also, loyalty, altruism, and obedience are correlated with procedural justice (R^2^ = 0.69) and loyalty and self-development are correlated with distributional justice (R^2^ = 0.89). A correlation was also detected between interactional justice and organizational identity (R^2^ = 0.89). The findings of the study could serve to identify the factors contributing to the creation and recreation of organizational identity, citizenship behavior and justice among nurses, to promote the performance of the organization, and to achieve organizational goals.

## 1. Introduction

Nursing is a job which involves close contact with people. Nurses are responsible for the health and lives of humans. The wide range of the responsibilities and the interdisciplinary nature of this job create numerous difficulties for the professionals. These difficulties include the conflict between the role and personality, little familiarity with colleagues, weak relations among nurses, various career responsibilities, various attitudes towards salary and benefits, and their attitudes toward their collective selves. Like other organizations, hospitals need to recruit personnel who contribute to achieve organizational goals based on the principles and missions of the organization. The speed and accuracy of achieving goals and effective productivity relies on conscientious staff who feels a close connection between their personal and organizational goals. Moreover, hospitals, as service-providing organizations, need to encourage investment and improve their reputation. This would be impossible in the absence of collaborative staff, particularly nurses (Thomas, 2007).

One of the most important advantages of taking into account organizational identity, justice, and citizenship behavior would be improving the performance and effectiveness of the organization and such benefits as a sense of attachment, organizational cooperation satisfaction, and fewer career moves.

Organizational citizenship behavior (OCB) refer to behaviors that are intended to help coworkers, the supervisor or the organization and include acts such as assisting coworkers, trying to improve workplace morale, volunteering for work that is not part of the job description, speaking highly of the organization to outsiders as well as suggesting improvements in the functioning of the organization (Crede et al., 2007). The findings of a study showed that existence of OCB in Iranian nurses are essential in developing patient-oriented behavior (Daraghi, Alirezaei, & shaham, 2012). OCB reduces the need for monitoring and time-consuming for scheduling, problem solving and make organizational practice more effective (Ackfeldt & Coote, 2005). Nurses are at the first-line of care and they play many roles in hospitals such as manager, leader, and teacher (Arbabisarjou, 2011). OCB enhance job satisfaction among nurses’ personnel. Nurses’ OCB have a positive significant influence on job satisfaction (Daraghi, Alirezaei, & Shaham, 2012).

Organizational identity guarantees that the staff is moving toward achieving the goals of the organization; in this sense, organizational identity is an essential concept in organizational behavior. It increases the probability that individuals stay in the organization and boost their cooperation with colleagues. In addition, organizational identity could be regarded as a mechanism of encouragement. Given the concept of identity, creation or recreation of organizational identity in hospitals can bring considerable influence to the personnel and direct them toward organizational activities so that both organizational goals and personal satisfaction is achieved. Therefore, the theory of organizational identity view identity as a feature of the organization which regards members as pivotal, stable, and internal qualities and improve the concept of organization in the mind of the staff (George & Jones, 2008).

Identity refers to the essential concept that the individuals have of their selves (Pratt et al., 2006). According to Empson (2004), identity involves answering such questions as “who am I?” or “what are we?”. Organizational identity includes an understanding shared by the collectivity. The “collectivity” in a hospital can be nurses, staff, or other groups. It should be noted that the organizational identity of the collectivity of nurses might be totally different from other personnel. “Shared understanding” is a conceptual feature. It is a set of concepts and beliefs about the organization held by the members or a type of Gestalt feature arising from group activities (Pratt et al., 2006).

Simply put, nurses as members of collectivity hold a common understanding of “what are we?”. This understanding which focuses on a pivotal and stable feature is referred to as the nurses’ organizational identity. Those pivotal and stable features can determine whether organizational identity is constructive and in line with the goals of the hospital or a destructive one which is against organizational missions.

One of the most stable bodies of literature regarding organizational studies is the perception of justice inherent in the organizational procedures and behaviors elicited from the staff. Liponnen et al. (2004) stated that the perception of justice held by staff is a vital factor in certain career behaviors. Fair and just consequences and procedures are considered valuable since they can bring positive consequences for nurses. According to another explanation, the degree of motivation underlying fair and just procedures forms the basis of humans’ tendency toward justice. Generally, justice has three types (McDowall & Fletcher, 2004):

a) Distributive Justice: it refers to the concerns of staff regarding resource and consequence distribution. McDowall and Fletcher (2004) states that distributive justice demonstrates the individual’s perception of the degree of justice in distributing and allocating resources and rewards (Rezaieyan, 2005). Comparing themselves with others, nurses come to understand whether they have achieved what they deserve. It should be noted that the definitions provided for distributive justice focus on the economic or instrumental dimensions of the fairness of the consequences. In other words, nurses determine the degree of attempt and what they deserve to achieve based on economic consequences.
b) Procedural Justice: it deals with the individuals’ impression of the fairness of decision-making procedures to pay for their services rather than with the real distribution of incomes (Folger & Konovski, 1989). When procedural justice is weak in a hospital, the nurses think that their efforts and time and energy they put to work are not fairly evaluated or that their rewards are not proportionate to their efforts to address the issues of the society. When nurses are poorly paid, procedural justice is more clearly perceived and highlighted. According to research, employees with high or medium levels of payment consider their salaries fair whether or not the processes are procedurally fair. On the other hand, poorly paid employees regard their salaries as fair only when their payments are the outcomes of procedural justice (Brockner et al., 1995).
c) Interactional Justice: it deals with the fairness of interpersonal relations within organizational procedures (McDowall & Fletcher, 2004). It is an important variable in identifying attitudes and behaviors of employees in response to being unemployed, making decisions regarding budgets, recruiting processes, and providing services to customers (Hornstein, 1996).

Based on the theory of organizational justice, it could be predicted that employees react to the presence or absence of organizational justice. One reaction is increases or decreases in outputs. If the nurses feel that the organization is not procedurally fair, they experience negative tension which decreases their outputs and cooperation within the organization. Conversely, if the nurses observe procedural justice in the hospital, they are motivated to increase their outputs and cooperation by performing helping behaviors such as organizational citizenship behaviors. In this case, according to the theory of Van Dyne and Graham (1994), there is an arbitrary relation between the employees and the organization (Noami & ShekarShekan, 2004). Findings of a study demonstrated that job satisfaction and organizational justice have significant effect on the nurses citizenship behaviors (C. S. Chang & H. C. Chang, 2010).

The third variable examined in the present study and is closely correlated with the other two refers to those measures which are voluntary and are not explicitly rewarded by formal organizational reward systems (Smith et al., 1983). Nevertheless, Organ (1997) redefined the construct of organizational citizenship behavior and introduced it as a voluntary behavior which is typically not rewarded but improves the functioning of the organization. He stated that organizational citizenship behavior might be identified and rewarded during performance evaluation. Past research introduces major attitudinal factors including perception of justice, identity, and support as the antecedents of organizational citizenship behavior.

Organ (1990) argues that a perception of fairness plays an important role in promoting organizational citizenship behavior. Recently, scholars have focused on those employee actions which are beyond the formal requirements of the job. A literature review demonstrates nearly 30 various potential behaviors which overlap and could be classified into 7 categories (Podsakoff et al., 2000).

1) Altruism: it is an important form of organizational citizenship behavior which is practically taken into account by any individual who works in the organization. Conceptually, it is referred to as providing voluntary help to others and preventing from career problems and conflicts and includes “helping absent employees”, “helping employees with heavy workloads”, “giving guidance to new employees even those who do not ask”, “always being prepared to help others”, and “not taking advantage of other people’s honesty” (Organ et al., 2006).
2) Sportsmanship: it includes tolerating inevitable work problems without complaining and “always highlighting positive sides of the job”, “not blowing problems out of proportion”, “not ascribing blames to the organization or others”, “not assigning their own work to others by flattery” (Organ, 1990).
3) Civic virtue: it is characterized by voluntary behaviors on the part of employees which demonstrate their responsible cooperation, involvement and deep concern about the life of the organization. It includes high levels of interest and full commitment to the organization through “presence in all meetings”, “expressing personal opinions regarding the strategies adopted by the organization”, “monitoring environmental opportunities and threats” (Podsakoff et al., 2000).
4) Conscience: voluntary behaviors on the part of employees which are beyond job requirements regarding presence, obedience, rules and regulations, taking leaves, and so forth. It includes “not taking excessive leaves”, “observing rules and regulations even when they are not monitored”, “believing in hard work to make a decent living” (Organ et al., 2006).
5) Loyalty: it involves a sense of identity and totally obeying the manager and going beyond the individuals’ group interests which include “seriousness in advertising for the organization”, “supporting the organization against others”, and “remaining committed under any circumstances” (Organ et al., 2006).
6) Obedience: it is regarded as a dimension of organizational citizenship behaviors since not every employee totally obeys the organizational rules and regulations, although they are expected to. On this basis, an employee who follows all rules and regulations even when nobody is observing is considered a good citizen (Podsakoff et al., 2000).
7) Self-development: it involves voluntary activities on the part of employees to improve skills, knowledge, and job abilities as well as keeping with the latest trends in the career. This dimension is conceptually the most general one and refers to individual efforts of the members to improve productivity (Van Dyne & Graham, 1994).

**Figure 1:**
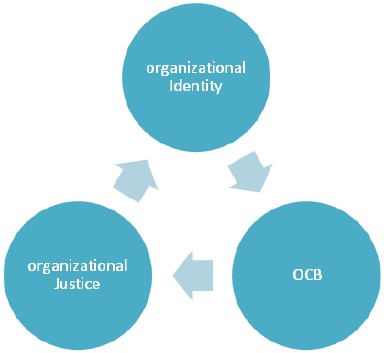
Conceptual model for OCB, organizational identity, and organizational justice

Figure 1 demonstrates the conceptual model of the study.

## 2. Methods and Materials

This research is a descriptive-correlative study. The population included all nurses working at hospitals affiliated to administry of health, treatment and medical education in Shahre-Kord (Iran) 2009.

Its purpose was to investigate the relationships among organizational identity, justice, and citizenship behavior among the nurses working at hospitals. The methodology is descriptive-correlative and the population consists of all nurses working at hospitals (N = 486) in 2009. The sample was selected through random sampling. The sample size was initially 165 but due to lack-of-response possibilities, the size increased to 175 out of which 168 were returned (return rate = 96.66). Three questionnaires were used to collect data:

a) Cheney’s Organizational Identity Questionnaire (1982): with 25 questions, it is highly valid with 94% reliability. The answers are scaled on a 5-degree Lickert scale ranging from absolutely agree to absolutely disagree.
b) Beugre’s Organizational Justice Questionnaire (1998). It contains 35 questions scaled on a 5-degree Lickert scale ranging from absolutely agree to absolutely disagree and evaluates distributional justice, procedural justice, and interactional justice, respectively.
c) The Organizational Citizenship Behavior Questionnaire: it is a modified 32-question scale devised by Smith et al. (1983) and is an adaptation of Organ (1997). Nevertheless, due to the comprehensiveness of the questions and the size of the questionnaire, a number of modifications was made to the questionnaire under the supervision of behavioral scientists and its reliability was calculated using Cronbach’s alpha, which was 0.89. It is devised on a Lickert scale ranging from absolutely agree to absolutely disagree.

## 3. Findings

The findings are demonstrated in Tables 1 to 5.

**Table 1:** Regressions among OCB dimensions and organizational Identity, Stepwise Method

	Model	R^2^	Standardized coefficients	t	p-value
			Beta		
	1				
Altruism		0/41	0/78	4/01	0/00

	2				
Altruism		0/512	0/58	3/22	0/00
Civic Virtue			0/2	1/5	0/00
	3				

Altruism		0/582	0/58	3/22	0/00
Civic Virtue			0/12	1/23	0/00
Loyalty			0/08	1/1	0/00
	4				

Altruism		0/612	0/58	3/22	0/00
Civic Virtue			0/12	1/23	0/00
Loyalty			0/05	1/2	0/00

Self-development			0/03	1/2	0/00

**Table 2:** Regressions among OCB Dimensions and Distributional Justice, Stepwise Method

	Model	R^2^	Standardized coefficients	t	p-value
			Beta		
	1				
Loyalty		0/51	0/81	9/48	0/002

	2				
Loyalty		0/71	0/61	8/1	0/002
Obedience			0/2	4/1	0/006

**Table 3:** Regressions among OCB Dimensions and Procedural Justice, Stepwise Method

	Model	R^2^	Standardized coefficients	t	p-value
			Beta		
	1				
Loyalty		0/54	0/97	15/29	0/000

	2				
Loyalty		0/62	0/57	16/1	0/00
Altruism			0/4	2/18	0/00

	3				
Loyalty		0/69	0/57	16/1	0/00
Altruism			0/3	2/4	0/00
Obedience			0/1	1/2	0/006

**Table 4:** Regressions among OCB Dimensions and Interactional Justice, Stepwise Method

	Model	R^2^	Standardized coefficients	t	p-value
			Beta		
	1				
Loyalty		0/86	0/000	19/317	0/000

	2				
Loyalty		0/89	0/00	20/40	0/00
Self-development			0/00	3/18	0/00

**Table 5:** Regressions among Organizational Justice Dimensions and Organizational Identity, Stepwise Method

	Model	R^2^	Standardized coefficients	t	p-value
			Beta		
	1				
Interactional Justice		0/75	0/68	12/13	0/000

Table 1 demonstrated the first hypothesis:

The dimensions of organizational citizenship behavior (altruism, civic virtue, conscientiousness, sportsmanship, loyalty, obedience, and self-development) and organizational identity are correlated.

Of all dimensions incorporated into the model, three dimensions (sportsmanship, obedience, and conscientiousness) were excluded and altruism, loyalty, and self-development remained in the model (P = 0.000). These dimensions are able to predict 0.612 changes in organizational identity. In other words, altruism (P = 0.000, β = 0.58), civic virtue (P = 0.000, β = 0.12), loyalty (P = 0.000, β = 0.05), and self-development (P = 0.000, β = 0.03) are correlated with organizational identity.

Table 2 demonstrates the results of the examination of the second hypothesis:

The dimensions of organizational citizenship behavior (altruism, civic virtue, conscientiousness, sportsmanship, loyalty, obedience, and self-development) and distributional justice are correlated.

Of all seven OCB dimensions incorporated into the model, 5 dimensions (altruism, sportsmanship, virtue, conscientiousness, and self-development) were excluded and loyalty and obedience remained (P = 0.000). They are able to predict 0.71 of the changes in distributional justice. In other words, loyalty (P = 0.002, β1 = 0.61) and obedience (P = 0.006, β2 = 0.2) are correlated with distributional justice.

Table 3 demonstrates the third hypothesis:

The dimensions of organizational citizenship behavior (altruism, civic virtue, conscientiousness, sportsmanship, loyalty, obedience, and self-development) and procedural justice are correlated.

Of all seven OCB dimensions incorporated into the model, 4 dimensions (sportsmanship, virtue, conscience, and self-development) were excluded and loyalty, altruism, and obedience remained in the model (P = 0.000). They can predict 0.69 of the changes in procedural justice. In other words, loyalty (P = 0.00, β1 = 0.57), altruism (P = 0.00, β2 = 0.3), and obedience (P = 0.006, β3 = 0.1).

Table 4 demonstrates the fourth hypothesis:

The dimensions of organizational citizenship behavior (altruism, civic virtue, conscientiousness, sportsmanship, loyalty, obedience, and self-development) and interactional justice are correlated.

Of all seven dimensions of OCB incorporated into the model, 5 dimensions (altruism, sportsmanship, virtue, conscience, and obedience) were excluded and loyalty and self-development remained (P = 0.000). They can predict 0.89 of the changes in interactional justice. In other words, loyalty (P = 0.00, β1 = 0.57) and self-development (P = 0.00, β1 = 0.3) are correlated with interactional justice.

Table 5 demonstrates the results of the 5th hypothesis:

The dimensions of organizational justice (distributional, procedural, and interactional) and organizational identity are correlated.

Distributional and procedural justice were excluded from them model and interactional justice remained. It can predict 0.75 of the changes in organizational identity. In other words, interactional justice and organizational identity are correlated (P = 0.00, β = 0.68).

## 4. Conclusion

The findings of the study revealed correlations between organizational identity and interactional justice and organizational citizenship behavior as well as between distributional justice and procedural justice and organizational citizenship behavior. Correlations between identity and OCB are demonstrated in Haigh and Pfao (2006). Desilvia, Sabag, and Ashten (2006) demonstrated the correlation between justice and CBO. Dicremer (2005) revealed the correlation between identity and organizational justice. Na’ami and Shekarshekan demonstrated the correlation between justice and CBO. Logically, these variables are directly correlated. Employees who perceive demonstrations of justice in organizational procedures and outcomes regard the organizational identity as one which is in line with and supports their personal identity, consider the organization’s success equal to their own, and try to promote the organization. Consequently, they go beyond merely fulfilling duties and demonstrate civil behaviors. The demonstration of such meta-functional behaviors results in efforts to be more effective, efficient, and productive and creates a cooperative atmosphere. This atmosphere creates confidence in the organization and longer cooperation with the organization. This cooperation makes a sense of value an essential part of the identity of the organization and creates justice and citizenship behavior. This secure atmosphere is in line with a perception of justice and prevents it from being damaged. In such organizations, meta-functional behaviors are indispensable parts of the employees’ behaviors and this cycle eventually leads in improved productivity.

### 4.1 First Hypothesis

The results of testing the first hypothesis, demonstrated in Table 1, revealed that altruism, virtue, loyalty, and self-development are correlated with organizational identity. Altruism is referred to as a set of behaviors the nurses do to their patients and colleagues. It involves nurses helping their colleagues fulfill their tasks and at difficult times such as absent colleagues or unpredicted events, they help each other. The significant correlation between identity and altruism reveals that the nurses’ organizational identity, which is the answer to the question “who are we?”, is interrelated with this factor. Civic virtue, which reflects voluntary behaviors to improve the organization, is related to the perception of the nurses of organizational identity. Therefore, allowing nurses to freely express their ideas and monitoring to create a trustable environment on the part of the managers can help nurses have a better understanding of their collective selves. Loyalty and self-development, which refer to the employees attempting to improve personally, involves the fact that if suitable grounds are provided for nurses to improve personally and professionally, it can affect the answer to the question “who are we?”.

### 4.2 Second, Third, and Fourth Hypotheses

The findings of the second, third, and fourth hypotheses, represented in Tables 2, 3, and 4, reveal that the dimensions of CBO are correlated with organizational justice. This correlation is especially considerable between loyalty and obedience with various forms of justice. It implies that nurses with higher levels of loyalty and obedience believe that some forms of justice exist in the organization. In other words, creating justice in the current procedures of the organization or in dealing with the nurses and distributing benefits and rewards can lead to senses of loyalty and obedience in nurses. This is in line with the findings of Ball et al. (1994), Tayler (2003), and Ashja’ (2007), who state: the direct impact of distributional justice on individuals’ reaction in the workplace is affected by procedural justice. In fact, there is convincing evidence that the impacts of procedural justice are strongly observed when the outcomes are not favorable. While favorable outcomes can satisfy individuals in general, unfavorable ones require a greater effort to explain and focus people’s attentions on procedures adopted to achieve goals. On this basis, with unfavorable outcomes, procedural justice has a great impact on the individuals’ attitudes toward the organization (De cremer, 2005).

### 4.3 Fifth Hypothesis

The findings of the fifth hypothesis, demonstrated in Table 5, reveal that interactional justice is correlated with organizational identity. Interactional justice refers to the perception of the fairness of interpersonal relations within the organization. If nurses feel that there is justice in their interactions with the authorities, they remain loyal to the organization and act toward achieving organizational goals. As mentioned before, identity is a shared understanding among nurses if its antecedents, justice and CBO, are present in the organization. Few Iranian studies have explained the correlation between justice and identity but the findings of Haigh and Pfao (2006), Desilvia, Sabag, and Ashten (2006), and De Cremer (2005) are in line with those of the present study.

## References

[ref1] Ackfeldt Al, Coote L. V (2005). A study of organizational Behaviors in a retail setting. JBR.

[ref2] Arbabisarjou A, Sullivan J, Eleanor, Gallend G (2011). Practical leadership and management in nursing.

[ref3] Ashja’ A (2007). The Correlations among Organizational Justice Antecedents.

[ref4] Ball G. A, Trevino L. K, Sims H. P (1994). Just and unjust punishment: influence on subordinate performance and citizenship. Academy of Management Journal.

[ref5] Beugre C. D (1998). Implementing business process reengineering: The role of organizational justice. Journal of Applied Behavioral Science.

[ref6] Brockner J, Wiesenfeld B. M, Martin C. L, Brockner J, Wiesenfeld B. M, Martin C. L (1995). Decision frame, procedural justice, and survivors’ reactions to job layoffs. Organizational Behavior & Human Decision Processes.

[ref7] Chang C. S, Chang H. C (2010). Moderating effect of nurses’ customer-oriented perception between OCB and satisfaction. WJN.

[ref8] Cheney G (1982). Organizational identification as process and product: A field study.

[ref9] Crede M, Chernyshenko O. S, Stark S, Dalal R. S, Bashshur M (2007). Job satisfaction as meditor: An assessment of job satisfaction’s position within the nomological network. Journal of Occupational and Organizational Psychology.

[ref10] Daraghi H, Alirezaie S, Shaham G (2012). Organizational citizenship behaviors among Iranian nurses.

[ref11] De Cremer D (2005). Procedural and distributive justice effects moderated by organizational identification. Journal of Managerial Psychology.

[ref12] Desivilya H. S, Sabag Y, Ashton E (2006). Prosocial tendencies in organizations. International Journal of Organizational Analysis.

[ref13] Empson L (2004). Organizational identity change, managerial regulation and member identification in an accounting firm acquisition. Accounting Organizational and Society.

[ref14] Folger R, Konovsky M. A (1989). Effects of procedural and distributive justice on reactions to pay raise decisions. Academy of Management Journal.

[ref15] George J. M, Jones G. R (2008). Organizational spontaneity in context. Human Performance.

[ref16] Haigh M. M, Pfau M (2006). Bolstering organizational identity, commitment, and citizenship behaviors through the process of inoculation. International Journal of Organizational Analysis.

[ref17] Hornstein H. A (1996). Brutal Bosses and Their Prey.

[ref18] Lipponen J, Olkkonen M, E, Myyry L (2004). Personal value orientation as a moderator in the relationships between perceived organizational justice and its hypothesized consequences. Social Justice Research.

[ref19] McDowall A, Fletcher C (2004). Employee development: an organizational justice perspective. Personnel Review.

[ref20] Noami A, Shekarshekan H (2004). Simple and Multiple Correlations of Organizational Justice with Job Satisfaction in Employees of an Industrial Company. Journal of Shahid Chamran University of Ahvaz, Department of Educational Sciences.

[ref21] Organ D. W (1990). The motivational basis of organizational citizenship behavior. Research in Organizational Behavior.

[ref22] Organ D. W (1997). Organizational citizenship behavior: It’s construct clean-up time. Human Performance.

[ref23] Organ D.W, Podskoff M. P, Machkenzie B. S (2006). Organization citizenship behavior, is nathre, antecedent and consequences.

[ref24] Podssakof P. M, McKenzie S. B, Paine J. B, Bacharach D. G (2000). Organizational citizenship behavior: a critical review of theoretical and empirical literature and suggestions for future research. Journal of Management.

[ref25] Pratt M. G, Rockmann K, Kaufman J (2006). Constructing Professional Identity: The Role of Work and Identity Learning Cycles in the Customization of Identity among Medical Professionals. Academy of Management Journal.

[ref26] Rezayean A (2005). Justice Expectations and Justice in Organization.

[ref27] Smith C. A, Organ D. W, Near J. P (1983). Organizational citizenship behavior: its nature and antecedents. Journal of Applied Psychology.

[ref28] Taylor A. G. W (2003). Justice as a Basic Human Need. New Ideas in Psychology.

[ref29] Thomas (2007). What is organizational identity and what have blogs to do with this?. www.Stylewalker.net.

[ref30] Van dyan Graham J. W, Dienesch R. M (1994). Organizational citizenship behavior, construc readefinition, measurement and validation. Academy of Management Journal.

